# The Impact of Endovascular Repair of Ruptured Abdominal Aortic Aneurysm on the Gastrointestinal and Renal Function

**DOI:** 10.1155/2014/178323

**Published:** 2014-01-29

**Authors:** R. R. Makar, S. A. Badger, M. E. O'Donnell, C. V. Soong, L. L. Lau, I. S. Young, R. J. Hannon, B. Lee

**Affiliations:** Vascular and Endovascular Surgery Unit, Belfast City Hospital, Lisburn Road, Belfast BT9 7AB, UK

## Abstract

*Introduction*. Systemic effects of ruptured abdominal aortic aneurysm (rAAA) may be altered by the mode of surgery. This study aimed to determine systemic effects of endovascular aneurysm repair (EVAR) compared to open repair (OR). *Patients and Methods*. Consecutive patients with rAAA were repaired by OR or EVAR according to computerised tomographic (CT) findings. Renal function was monitored by estimated glomerular filtration rate (eGFR), serum urea and creatinine, and urinary albumin creatinine ratio (ACR). Hepatic function was assessed postoperatively for 5 days. Intestinal function was determined by the paracetamol absorption test. Intestinal permeability was assessed by urinary lactulose/mannitol ratio. *Results*. 30 rAAA patients were included. Fourteen had eEVAR and sixteen eOR. Serum urea were higher in eOR, while creatinine was similar between groups. Hepatic function showed no intergroup difference. Paracetamol absorption was increased in eEVAR group at day 3 compared to day 1 (*P* = 0.03), with no similar result in eOR (*P* = 0.24). Peak lactulose/mannitol ratio was higher in eOR (*P* = 0.03), with higher urinary L/M ratio in eOR at day 3 (*P* = 0.02). Clinical intestinal function returned quicker in eEVAR (*P* = 0.02). *Conclusion*. EVAR attenuated the organ dysfunction compared to open repair. However, a larger comparative trial would be required to validate this. The clinical trial is registered with reference number EUDRACT: 2013-003373-12.

## 1. Introduction

The gastrointestinal tract is a barrier between the body and the potentially harmful intraluminal pathogens and antigens [1]. It has been implicated in the development of systemic inflammatory response syndrome (SIRS), through bacterial translocation, which initiates or sustains the septic state of the host leading to multiple organ failure [2]. The mucosal barrier consists of mechanical, biological, chemical, and immunological components, with the epithelial lining being the most important [3].

During abdominal aortic aneurysm (AAA) surgery the intestine is subjected to ischaemia and reperfusion. In open repair, there is direct trauma and desiccation, with ileus being postoperatively common [4]. Surgical trauma triggers the release of inflammatory mediators, which inhibit bowel function and stimulate sympathetic neural activities [5]. Although postoperative transmural ischaemic bowel infarction is rare, subclinical mucosal ischaemia is not uncommon [6, 7]. Traction of the mesentery and clamping/unclamping of the aorta causes the release of vasoconstrictive mediators thus compromising blood flow to the bowel [8, 9].

Increased small bowel permeability postoperatively, with portal endotoxaemia, has been demonstrated [10]. An increase in intestinal permeability occurs following ruptured aneurysm (rAAA) repair and in trauma patients with significant severity [11, 12]. During hypotension, blood flow may be shunted from the splanchnic circulation to the vital organs [13]. The enterocytes are particularly susceptible to ischaemia injury because of the counter-current exchange mechanism [14]. The oxygen tension at the tip of the villus is lower compared to the base even in normal situations. This discrepancy in oxygen tension will be exaggerated in times of hypotension [15, 16].

Ischaemia-reperfusion injury to other organs may occur during AAA surgery. Edrees et al. showed that ischaemia-reperfusion injury to the lower limbs leads to an increase in intestinal permeability in patients following infrainguinal lower limb bypass surgery [16].

The aim of this study was to investigate the effect of emergency endovascular aneurysm repair (eEVAR) on various organ functions compared to emergency open repair (eOR).

## 2. Patients and Methods

The details of the overall project have been described before [17]. However, in brief, patients presenting to the vascular surgery unit with an infrarenal ruptured AAA were recruited after written informed consent from patient or next of kin. All patients underwent preoperative CT scan and anatomical details were assessed for suitability for endovascular repair by the on-call consultant vascular surgeon and interventional radiologist. Exclusion criteria were no extravasation of blood on CT scan, juxtarenal aortic aneurysm, dementia, refusal to participate, or chronic renal failure requiring haemodialysis. Patients who died within 2 hours of repair were also excluded from further analysis. Midline transperitoneal open repair was performed under general anaesthesia. Endovascular repair was performed under local anaesthesia, with a subsequent fem-fem crossover under general anaesthesia since an aortouni-iliac device was deployed. Demographic and perioperative details of the study group have already been reported [17].

Renal failure was defined as a persistent or progressive elevated serum creatinine concentration ≥200 *μ*mol/L that required haemofiltration or was associated with urine output <500 mL/24 hours in the absence of dehydration. Paralytic ileus was defined as absence of bowel sounds and failure to pass flatus or bowel motion for more than 4 days. Renal function was assessed using serum urea and creatinine and urinary albumin creatinine ratio. An estimation of glomerular filtration rate (eGFR) was made using an online-calculator derived from the UK CKD eGuide on the Renal Association website [18]. This derives an eGFR value based on the patient's serum creatinine, sex, age, and race. Hepatic function was assessed using serum bilirubin, aspartate transaminase (AST), gamma-glutamyl transaminase (*γ*GT), and alkaline phosphatase (ALP). All of these were measured preoperatively and postoperatively daily until day 5 following surgery.

Intestinal activity was determined by the presence of normal bowel sounds, passage of flatus, and tolerance to oral or nasogastric feeding [19]. Measurements of gastric motility were by the paracetamol absorption test. Intestinal permeability was assessed by urinary lactulose/mannitol ratio on the 1st, 3rd, and 5th postoperative days. The retest urinary sample was collected after 6 hours of fasting as a baseline measurement of urinary sugar content. Thereafter, patients were given 10 g of lactulose and 5 g of mannitol dissolved in 100 mls of water orally or via the nasogastric tube. All urine was collected for 6 hours. The total volume of the collected urine was recorded and a 10 mls sample was stored at −80°C until analysis.

### 2.1. Statistical Analysis

Continuous variables were expressed as mean (± standard deviation). Correlations were calculated by Spearman's rank correlation coefficient test between organ function measurements and operative parameters. The latter included preoperative systolic blood pressure, Hardman's score, operative time, intraoperative blood loss, packed cell transfusion, platelet transfusion, and total intravenous fluid administered. While *P* value of <0.05 was considered as borderline significant, results were only regarded as significant if the *P* values fell below 0.01, to allow for the effect of multiple comparisons.

## 3. Results

### 3.1. Patient Characteristics

During the two-year study period, 40 consecutive rAAA patients who reached the hospital alive were considered for the study, of which 10 were excluded. Two patients had suffered a cardiac arrest on arrival to the operating theatre, while a third patient died due to a severe myocardial infarction (MI) following aneurysm exclusion by deployment of the aorto-uni-iliac stent. One patient in each group were excluded because they were haemodialysis dependent preoperatively. The rest of the excluded patients (*n* = 5) had eOR because of unavailability of the facility or staff for eEVAR or lack of consent.

Thirty patients were included in the study. Fourteen patients had eEVAR and sixteen underwent an eOR. The average age for the eEVAR group was 72.2 (±6.2) years and the eOR was 71.4 (±7.0) years (Table 1). The male to female ratio was 6 : 1 in the eEVAR group and 7 : 1 in the eOR group. The baseline features were comparable for the two groups (Table 1). One eEVAR patient was on warfarin.

### 3.2. Anaesthesia and Intraoperative Details

In the eEVAR group, local anaesthesia with or without sedation was used in 11 patients. One patient had general anaesthesia, while two patients started with local anaesthesia but were converted to general anaesthesia during the femorofemoral crossover grafting. All patients who underwent eOR had standard general anaesthesia with endotracheal intubation.

All patients in the eEVAR group had an aorto-uni-iliac stent-graft (Talent, Medtronic Ave, Santa Rosa, CA) with the exception of one patient who had a bifurcated Talent stent-graft. There was no intraoperative conversion from eEVAR to eOR. Six eEVAR patients were found to have intraoperative type 1 endoleak, which was controlled with ballooning and Palmaz stent (Cordis Corporation, Miami, USA) in four patients, aortic cuff extension (Talent, Medtronic Ave, Santa Rosa, CA) in one patient, and both aortic cuff and Palmaz stent in another. No patient required an intra-aortic occlusion balloon. Two patients had an intraoperative type 2 endoleak, requiring no treatment and had gone on followup. In the eOR patients, 7 had a bifurcated Dacron graft while nine patients had a straight Dacron graft. All patients who had eOR had primary abdominal closure. The overall clinical outcomes and requirement for a higher level of care showed more favourable results for endovascular repair (Table 2), with these results having been reported previously [17].

### 3.3. Renal Functions

#### 3.3.1. Urea

**Figure 1:**
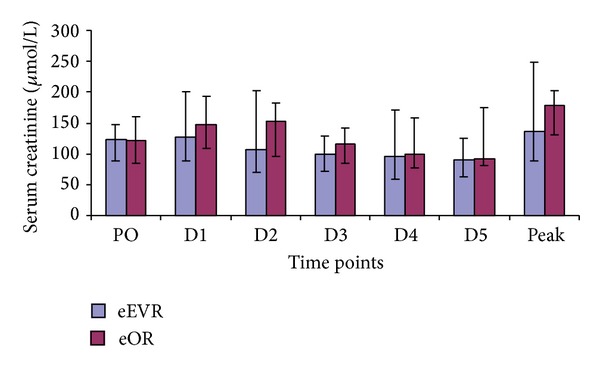
Serum urea levels in the eEVAR versus eOR groups at preoperatively (PO, *P* = 0.442), day 1 (D1, *P* = 0.429), day 2 (D2, *P* = 0.134), day 3 (D3, *P* = 0.240), day 4 (D4, *P* = 0.121), day 5 (D5, *P* = 0.062), and the peak (*P* = 0.328) expressed as median and IQR (Mann-Whitney *U* test).

Serum urea concentrations were consistently higher in the eOR group compared to the eEVAR (Figure 1). Both groups showed a significant increase in serum urea at day 1 (D1) compared to preoperative (PO) level (Table 3). The eOR group maintained significantly higher serum urea concentrations in all postoperative time points compared to PO, while the eEVAR group gradually decreased to no significance at D2 postoperatively. There was a weak correlation between peak serum urea and the volume of intraoperative red packed-cell transfused (*r* = 0.387; *P* = 0.03), but not with the other operative parameters.

#### 3.3.2. Creatinine

**Figure 2:**
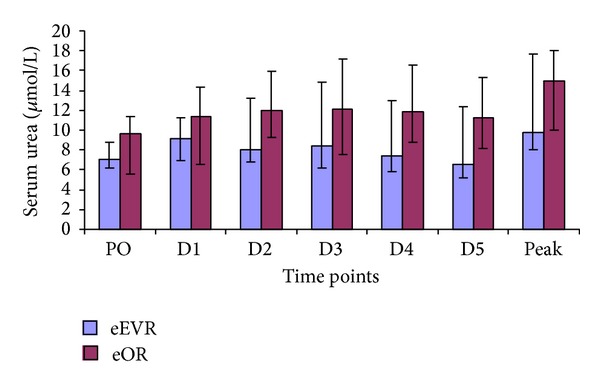
Serum creatinine concentrations in the eEVAR versus eOR groups at PO (*P* = 0.755), D1 (*P* = 0.493), D2 (*P* = 0.333), D3 (*P* = 0.645), D4 (*P* = 0.810), D5 (*P* = 0.549), and the peak (*P* = 0.467) expressed as median and IQR (Mann-Whitney *U* test).

Serum creatinine were similar throughout in the eEVAR and the eOR groups (Figure 2). However, a significant rise in serum creatinine was observed in the eOR group at D1 compared to the PO level (*P* = 0.02), with no difference within the eEVAR group between the PO and any of the postoperative time points (*P* > 0.05). There was a weak correlation between the peak serum creatinine concentration and the volume of intraoperative packed-cells transfused (*r* = 0.382; *P* = 0.03) and the Hardman's scores (*r* = 0.391; *P* = 0.03).

#### 3.3.3. Estimated Glomerular Filtration Rate (eGFR)

**Figure 3:**
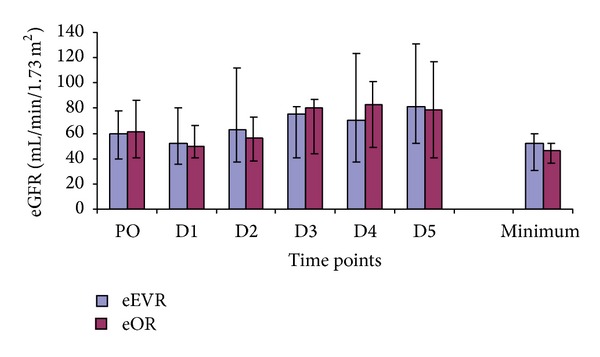
eGFR in the eEVAR versus eOR groups expressed as median and IQR (Mann-Whitney *U* test).

The eGFR was similar in both groups throughout (Figure 3). However, a significant decrease was observed in the eOR group at D1 compared to the PO level (*P* = 0.01), while there was a significant increase in eGFR within the eEVAR group at D5 compared to PO (*P* = 0.02). There was a negative correlation between minimum eGFR and the volume of intraoperative packed-cell transfused (*r* = −0.383; *P* = 0.03) and the Hardman's score (*r* = −0.614; *P* = 0.0001).

#### 3.3.4. Urinary Albumin-Creatinine Ratio (ACR)

**Figure 4:**
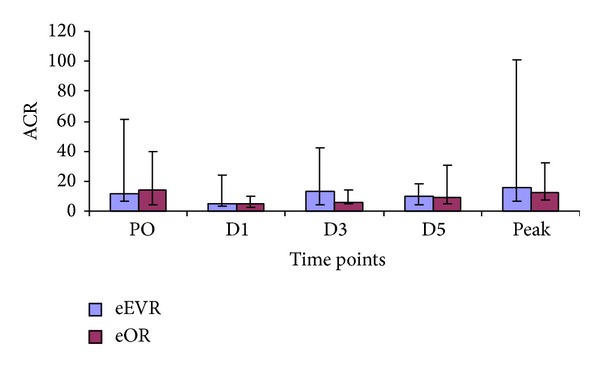
ACR levels in the eEVAR versus eOR groups preoperatively then, D2, D3, and D5 expressed as median and IQR. *P* = 0.047 for D3 compared to PO in the eOR group (Wilcoxon signed ranks test).

Urinary ACR was similar in the two groups throughout (Figure 4). However, a reduction in the ACR was observed on D3 (*P* = 0.047) compared to PO within the eOR group, while the eEVAR group showed no significant change at any of the time points. The only perioperative parameter that correlated with the peak ACR was Hardman's score (*r* = 0.451; *P* = 0.012).

### 3.4. Hepatic Function

#### 3.4.1. Serum Bilirubin

**Figure 5:**
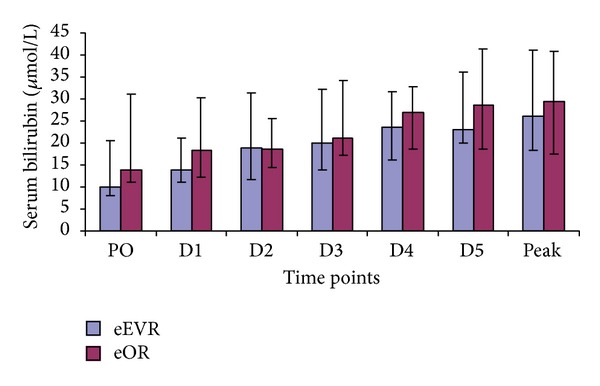
Serum bilirubin concentrations expressed as median and IQR (Mann-Whitney *U* test).

Serum bilirubin was similar in both groups at all time points (Figure 5). However, increased concentration was found within both groups compared to the PO levels (Table 4). Peak serum bilirubin did not correlate with any perioperative variables. 

#### 3.4.2. Serum Aspartate Transaminase (AST)

**Figure 6:**
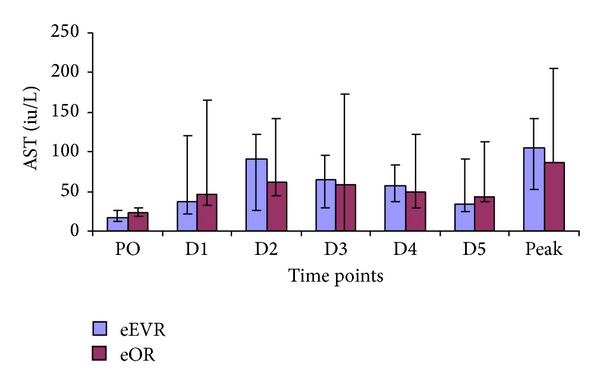
Serum AST expressed as median and IQR. At D1, D2, D3, D4, and D5, *P* < 0.01 (eOR group versus PO). At D2, D3, D4, and D5, *P* < 0.01. At D1 *P* < 0.05 (eEVAR group versus PO) (Wilcoxon signed ranks test).

AST concentration was similar in both groups at all time points (Figure 6). However, both groups showed significant rises in serum AST concentrations at all time points compared to PO (Figure 6). Operative times correlated with peak AST levels (*r* = 0.473, *P* = 0.008). Because of the anaesthetic difference between eOR and eEVAR the operative durations were correlated with the AST of each individual group. There was no correlation between the AST in the eEVAR group and the operative duration (*r* = 0.248, *P* = 0.39) while in the eOR group there was a significant correlation between the AST and the operative times (*r* = 0.662, *P* = 0.005). 

#### 3.4.3. Gamma Glutamyl Transaminase (*γ*GT)

**Figure 7:**
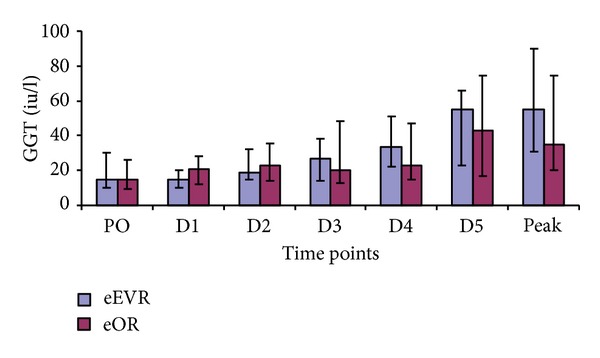
Serum *γ*GT expressed as median and IQR (Mann-Whitney *U* test). At D4, *P* = 0.005, (D4 versus PO). At D5, *P* = 0.003, (D5 versus PO) in eEVAR group. *P* = 0.04, (D4 versus PO) in eOR group (wilcoxon signed Ranks test).

No significant difference in *γ*GT was found between groups (Figure 7). Both groups showed a gradual rise in the *γ*GT concentrations compared to PO, reaching significance in D4 (*P* = 0.005) and D5 (*P* = 0.003) in the eEVAR group and D5 (*P* = 0.04) in the eOR group (Figure 7).

### 3.5. Intestinal Function

#### 3.5.1. Paracetamol Absorption Test for Gastric Motility

**Figure 8:**
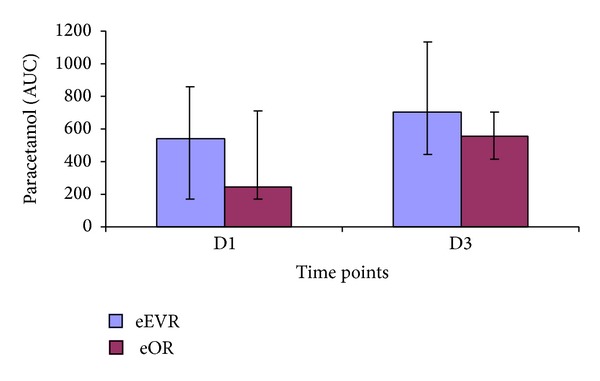
AUC_120_ for plasma paracetamol concentration expressed as median and IQR (*μ*g/mL/min). *P* = 0.03, D1 versus D3 in eEVAR group (Wilcoxon signed ranks test).

**Figure 9:**
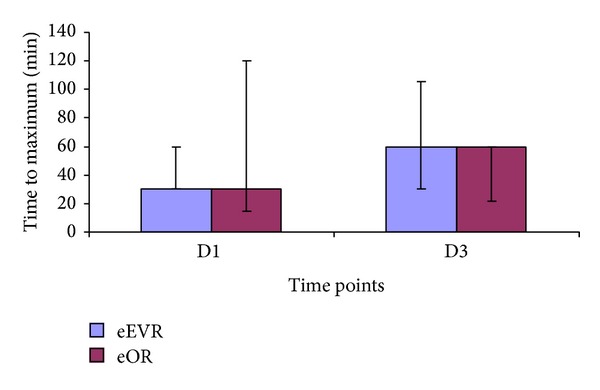
Time to reach maximum plasma paracetamol concentration at D1 (*P* = 0.95) and D3 (*P* = 0.37) expressed as median and IQR (Mann-Whitney *U* Test).

Paracetamol absorption increased in the eEVAR group at D3 compared to D1 (*P* = 0.03), but not in the eOR group (*P* = 0.24; Figure 8). However, there was no difference in the time to reach maximum between the groups on either day, which was 30 minutes on D1 and 60 minutes on D3 for both groups (Figure 9). There was a negative correlation between plasma paracetamol AUC at D1 postoperatively and the total volume of intraoperative fluid infusion (*r*_*s*_ = −0.482, *P* = 0.008), but not with any other perioperative parameters. 

#### 3.5.2. Lactulose-Mannitol Ratio (L/M Ratio) for Bowel Permeability

**Figure 10:**
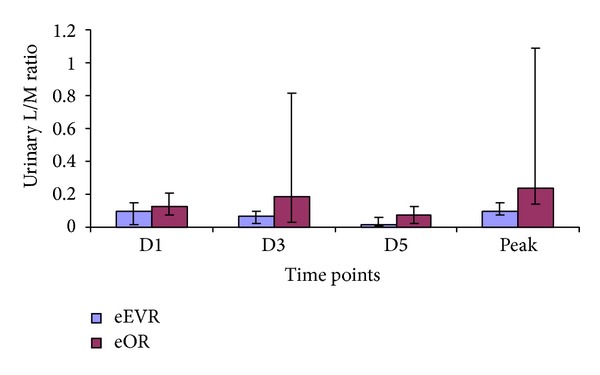
Lactulose/mannitol excretion ratio (median, IQR). At D3, *P* = 0.02 and at peak, *P* = 0.03 (eEVAR versus eOR) (Mann Whitney *U* test).

The eOR peak L/M ratio was higher compared to eEVAR group (0.2407 versus 0.0990; *P* = 0.03; Figure 10). There was also a higher urinary L/M ratio in the eOR group compared to eEVAR group at D3 (0.1854 versus 0.0633; *P* = 0.02). There was no significant change in the L/M ratio within the eOR or eEVAR groups; the reduction in ratio in the eEVAR group bordered on significance on D5 (*P* = 0.09) compared to D1 (Figure 10). Peak L/M ratio correlated with the intraoperative blood loss, packed-cell and platelets transfusion, and Hardman's score. 

#### 3.5.3. Bowel Sound, Flatus, and Oral Feeding

**Figure 11:**
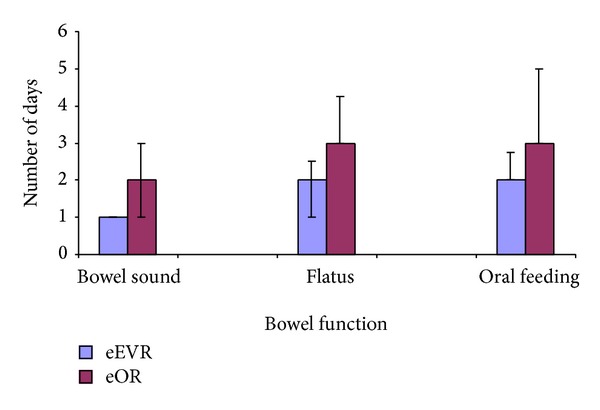
Bowel sound (*P* = 0.02), flatus (*P* < 0.01), and oral feeding (*P* < 0.01), eEVAR versus eOR (median, IQR; Mann-Whitney *U* test).

The presence of bowel sound, passing of flatus, and tolerance to oral feeding occurred earlier in the eEVAR group compared to the eOR group (*P* = 0.02, *P* = 0.003, and *P* = 0.004, resp.; Figure 11).

## 4. Discussion

The mortality of open repair for rAAA still ranges between 32% and 80%. However, eEVAR provides improved operative mortality rates with shorter hospital and ICU stays [20]. This present study investigated and compared the effect of eOR to eEVAR on the dysfunction of various organs. Clinical studies on elective AAA repair have shown the advantage of EVAR over OR in respect to postoperative renal, cardiac, and respiratory complications and the early return of bowel function [21, 22]. Transient isolated organ dysfunction may only be manifested as deterioration of its biochemical parameters without requiring organ support. Single organ dysfunction may create an additional strain on other compromised organs and is associated with mortality in patients with rAAA [23, 24].

Although not reaching significance, the present findings suggest that eOR has a negative impact on renal function in rAAA patients. Other studies reported renal impairment in one-third of the patients following open rAAA repair [25, 26]. Both haemodynamic derangement and lower torso ischaemia-reperfusion injury contribute to postoperative renal impairment following open AAA repair [27]. Conversely, Hinchliffe et al. observed renal impairment in 55% amongst the eEVAR group versus 8% in eOR group, perhaps due to atherosclerotic embolisation or contrast nephropathy in eEVAR [28]. The current study did not show a significant increase in serum creatinine postoperatively in the eEVAR group, perhaps due to the positive fluid balance achieved during the first 24 hours postoperatively.

The rise in bilirubin and AST in both groups is in keeping with other studies [29]. Splanchnic vasoconstriction in hypovolemic shock profound enough to cause renal impairment may also result in hepatocellular damage [29]. A study on elective open AAA repair observed an association between the duration of intraoperative hypotension and metabolic acidosis and the development of liver dysfunction [30]. In our study, the lack of inter-group hepatic enzymes and bilirubin difference could be due to the impact of haemorrhagic shock and the retroperitoneal haematoma in both groups. The correlation between the duration of surgery and AST in the eOR group implies that general anaesthesia medications could contribute to the hepatocellular injury in addition to ischaemia resulting from haemodynamic derangement secondary to loss of the abdominal wall tamponade effect and loss of sympathetic tone.

The paracetamol absorption in the eEVAR group indicates the return of gastric motility by the 3rd postoperative day. Postoperative paralytic ileus usually involves the stomach for the first 48 hours after open abdominal surgery [31]. However, gastric emptying can return to normal 18 hours after open elective AAA repair [32]. Arya et al. demonstrated that retroperitoneal AAA repair was associated with early return of gastric emptying function compared to transperitoneal approachdue to mesenteric traction and bowel manipulation [33]. While there was no intergroup difference, the eEVAR group gastric emptying was within the reported normal range by the 3rd postoperative day (median AUC 757.5 *μ*g/mL/min, IQR 507.7–1199.25). This delay in normalisation of gastric motility could be due to mechanical ventilation, sedatives, opioids, cytokine release and splanchnic hypoperfusion [34, 35]. Additionally, retroperitoneal haematoma and intra-peritoneal blood contribute to peritoneal irritation and adynamic ileus.

In the elective AAA repair sitting, EVAR patients have earlier return to oral intake compared to open repair [21, 22]. The delay in the eOR group to oral feeding and large bowel motility could be explained by secondary splanchnic hypoperfusion. The negative correlation between the paracetamol absorption at the day 1 and intraoperative intravenous fluid supports the association between volume overload and the postoperative ileus [36]. The higher intestinal permeability in the eOR group is suggestive of surgical approaches on its pathogenesis [37]. Furthermore, the eEVAR group in this study was observed to have a trend of decreased L/M ratio reaching its minimum at the 5th postoperative day, which is comparable to the normal range of L/M ratio in preoperative AAA patients reported from our unit and others [10, 37].

In the current study, both groups had mesenteric hypoperfusion as part of the global body ischaemia secondary to the haemorrhagic shock state. Thus the generalised ischaemia alone could not explain the difference in bowel permeability noted in both groups. Experimental animal studies have recognised that haemorrhagic shock with resuscitation is a total body ischaemia/reperfusion event [38]. Conversely, Roumen et al. found no difference in intestinal permeability between elective and emergency AAA patients [11]. They postulated that reperfusion rather than ischaemia is the main cause.

eOR subjects the patients to almost complete ischaemia of the pelvis and lower limbs during the aortic cross clamping while the eEVAR has incomplete lower limb ischaemia during the femorofemoral cross-over because of the perfusion of one internal iliac artery. Hence eEVAR could attenuate this ischaemia-reperfusion cycle. Lower limb ischaemia-reperfusion injury has been implicated in the increase in intestinal permeability in both human and in experimental studies [16, 39]. Additionally, lower limb ischaemia-reperfusion injury has been found to decrease splanchnic blood flow in experimental study [40].

Initial reports on eEVAR for rAAA suggested significant improvement in the operative mortality rate, ranging between 0% and 23% [41–44]. However, an international multicentre study showed a 30-day mortality of 37% in eEVAR compared to 39% for open repair. These rates may have been influenced by the heterogeneity between the centres [45]. Similarly, Hinchliffe et al. reported a 30-day mortality rate of 53% for both eEVAR and open repair [28]. However, two recent systematic reviews suggested that eEVAR in selected patients may have lower mortality and shorter hospital and ICU stays [20, 46]. Unfortunately, these two reviews were based on studies and case series that were nonrandomised, which could have led to a selection bias in favour of eEVAR.

The mortality rate of rAAA repair is also dependent on caseload for both eEVAR and open repair [47]. They reported a mortality of 45.9% following eEVAR in units that operate on <100 cases/4 years compared to 26% mortality in larger volume units. They also observed a similar relationship between hospital volume and open repair, with 51.5% for small volume hospitals versus 44.3% for large volume hospitals. Their overall reported mortality was 39.4% for eEVAR versus 47.7% for open repair of rAAA. Gerassimidis et al. reported a similar 30-day mortality of 39%, when they included in their study haemodynamically unstable patients (9 out of 23 patients) [48]. Their rate of major complications was 22%, which was much lower than that found by Hinchliffe et al. for eEVAR, 77% versus 80% for open repair [28].

Hechelhammer et al. reported abdominal compartment syndrome in 3 patients, with 4 patients requiring haemofiltration for acute renal failure, out of 37 patients after eEVAR. Their eEVAR mortality was 10.9% while the open repair was 35%. Type 2 endoleak was observed in 30.5% of their patients [44]. The cumulative risk of aneurysm related interventions was 35% at 2 years and 44% at 3 years; these were mainly for types 1 and 3 endoleaks. Lee et al. in a study comparing open repair versus eEVAR of rAAA, reported significantly less operation time, blood loss, and length of hospital stay in the latter group [49]. Similarly, Hinchliffe et al. showed less volume of blood lost and transfused in patients who had eEVAR compared to open repair [28]. Although the latter resulted in a potential physiological advantage for the eEVAR group, they observed more renal failure in this group. They suggested microembolization and radiocontrast as a potential cause for the renal compromise.

## Figures and Tables

**Table 1:** Preoperative patient characteristics.

Characteristics	eEVAR *n* = 14	eOR *n* = 16	*P* value
Mean (sd) age in years	72.2 (6.2)	71.4 (7)	0.75^†^
Male to female ratio	12 : 2	14 : 2	1.0^‡^
Risk factors			
Diabetes mellitus	0	0	—
Hypertension	11	12	1.0^‡^
Hyperlipidaemia	5	9	0.29^‡^
Comorbidities			
Ischaemic heart disease	8	11	0.70^‡^
Myocardial infarction	6	6	1.00^‡^
Carotid artery disease	4	0	0.07^‡^
PVD	2	0	0.20^‡^
COPD	2	3	1.00^‡^
Creatinine >150 *μ*mol/L	8	10	1.00^‡^
Systolic blood pressure at admission mean (sd)	101 (30.5)	108 (28.2)	0.56^†^
Hardman's score			
0	4	4	0.26^#^
1	5	2	
2	3	5	
3	2	4	
4	0	1	
Hardman's score 3 or above	2	5	0.39^‡^
Infrarenal abdominal aortic aneurysm diameter mean (sd) in mm	82 (17.7)	89 (15.8)	0.21^†^

Sd: standard deviation; PVD: peripheral vascular disease; COPD: chronic obstructive pulmonary disease. ^†^ *t*-test; ^‡^Chi-square test; ^#^Mann-Whitney *U* test.

**Table 2:** Intraoperative and postoperative outcomes.

	eEVAR (*n* = 14)	eOR (*n* = 16)	*P* value
Fluid management			
Blood loss (mLs)	862 (297–1183)	3767 (2275–6284)	<0.01
Packed cells transfused (units)	3 (2–4)	9 (5–11)	<0.01
Intraop IV fluid (mLs)	2250 (1500–3125)	4250 (3123–7500)	0.001

Postop care			
ICU admission	8	16	0.005
ICU LOS (hrs)	22 (0–78)	90 (48–168)	0.006
HDU admission	9	4	0.06
HDU LOS (hrs)	10 (0–25)	0 (0–3)	0.08
ICU/HDU LOS (hrs)	38 (9–102)	138 (49–168)	0.01
Hospital LOS	13.5 (9.7–22.2)	19 (9.2–29)	0.3

Complications			
Death	2	2	>0.99
Cardiac	2	2	>0.99
Respiratory	2	7	0.08
Renal	2	2	>0.99
Stroke	0	1	>0.99
GI ischaemia	1	1	>0.99

ICU: intensive care unit; HDU: high dependancy unit; GI: gastrointestinal.

**Table 3:** Within group comparison of serum urea levels at each postoperative time point as compared to the PO. The numbers in the table represent the *P* values (Wilcoxon signed ranks test).

	D1-PO	D2-PO	D3-PO	D4-PO	D5-PO
eEVR	0.01*	0.08	0.08	0.15	0.55
eOR	0.02*	0.01*	0.005*	0.006*	0.02*

**Table 4:** Within group comparison of serum bilirubin concentration at each postoperative time point as compared to the PO level. The numbers in the table represent the *P* values (Wilcoxon signed ranks test).

	D1-PO	D2-PO	D3-PO	D4-PO	D5-PO
eEVR	0.03	0.008	0.01	0.02	0.05
eOR	0.08	0.08	0.05	0.01	0.004

## References

[1] Saadia R, Schein M, MacFarlane C, Boffard KD (1990). Gut barrier function and the surgeon. *British Journal of Surgery*.

[2] Carrico CJ, Meakins JL, Marshall JC (1986). Multiple-organ-failure syndrome. *Archives of Surgery*.

[3] Baumgart DC, Dignass AU (2002). Intestinal barrier function. *Current Opinion in Clinical Nutrition and Metabolic Care*.

[4] Miedema BW, Schillie S, Simmons JW, Burgess SV, Liem T, Silver D (2002). Small bowel motility and transit after aortic surgery. *Journal of Vascular Surgery*.

[5] Mattei P, Rombeau JL (2006). Review of the pathophysiology and management of postoperative ileus. *World Journal of Surgery*.

[6] Fiddian-Green RG, Amelin PM, Herrmann JB (1986). Prediction of the development of sigmoid ischemia on the day of aortic operations. *Archives of Surgery*.

[7] Soong CV, Blair PHB, Halliday MI (1994). Bowel ischaemia and organ impairment in elective abdominal aortic aneurysm repair. *British Journal of Surgery*.

[8] Johnson WC, Nabseth DC (1974). Visceral infarction following aortic surgery. *Annals of Surgery*.

[9] Fry RE, Huber PJ, Ramsey KL, Fry WJ (1984). Infrarenal aortic occlusion, colonic blood flow, and the effect of nitroglycerin afterload reduction. *Surgery*.

[10] Lau LL, Halliday MI, Lee B, Hannon RJ, Gardiner KR, Soong CV (2010). Intestinal manipulation during elective aortic aneurysm surgery leads to portal endotoxaemia and mucosal barrier dysfunction. *EJVES Extra*.

[11] Roumen RM, van der Vliet JA, Wevers RA, Goris RJA (1993). Intestinal permeability is increased after major vascular surgery. *Journal of Vascular Surgery*.

[12] Roumen RM, Hendriks T, van der Ven-Jongekrijg J (1993). Cytokine patterns in patients after major vascular surgery, hemorrhagic shock, and severe blunt trauma: relation with subsequent adult respiratory distress syndrome and multiple organ failure. *Annals of Surgery*.

[13] Tokyay R, Zeigler ST, Traber DL (1993). Postburn gastrointestinal vasoconstriction increases bacterial and endotoxin translocation. *Journal of Applied Physiology*.

[14] Jodal M, Lundgren O (1986). Countercurrent mechanisms in the mammalian gastrointestinal tract. *Gastroenterology*.

[15] Landow L, Andersen LW (1994). Splanchnic ischaemia and its role in multiple organ failure. *Acta Anaesthesiologica Scandinavica*.

[16] Edrees WK, Lau LL, Young IS (2003). The effect of lower limb ischaemia-reperfusion on intestinal permeability and the systemic inflammatory response. *European Journal of Vascular and Endovascular Surgery*.

[17] Makar RR, Badger SA, O’Donnell ME, Loan W, Lau LL, Soong CV (2009). The effects of abdominal compartment hypertension after open and endovascular repair of a ruptured abdominal aortic aneurysm. *Journal of Vascular Surgery*.

[18] The UK Renal Association GFR online calculator. http://www.renal.org/eGFRcalc/GFR.pl.

[19] Ivatury RR, Simon RJ, Islam S, Fueg A, Rohman M, Stahl WM (1996). A prospective randomized study of end points of resuscitation after major trauma: global oxygen transport indices versus organ-specific gastric mucosal pH. *Journal of the American College of Surgeons*.

[20] Harkin DW, Dillon M, Blair PH, Ellis PK, Kee F (2007). Endovascular ruptured abdominal aortic aneurysm repair (EVRAR): a systematic review. *European Journal of Vascular and Endovascular Surgery*.

[21] Matsumura JS, Brewster DC, Makaroun MS (2003). A multicenter controlled clinical trial of open versus endovascular treatment of abdominal aortic aneurysm. *Journal of Vascular Surgery*.

[22] Lovegrove RE, Javid M, Magee TR, Galland RB (2008). A meta-analysis of 21178 patients undergoing open or endovascular repair of abdominal aortic aneurysm. *British Journal of Surgery*.

[23] Johnston KW (1994). Ruptured abdominal aortic aneurysm: six-year follow-up results of a multicenter prospective study. *Journal of Vascular Surgery*.

[24] Chen JC, Hildebrand HD, Salvian AJ (1996). Predictors of death in nonruptured and ruptured abdominal aortic aneurysms. *Journal of Vascular Surgery*.

[25] Gloviczki P, Pairolero PC, Mucha P (1992). Ruptured abdominal aortic aneurysms: repair should not be denied. *Journal of Vascular Surgery*.

[26] Noel AA, Gloviczki P, Cherry KJ (2001). Ruptured abdominal aortic aneurysms: the excessive mortality rate of conventional repair. *Journal of Vascular Surgery*.

[27] Boyle JR, Goodall S, Thompson JP, Bell PRF, Thompson MM (2000). Endovascular AAA repair attenuates the inflammatory and renal responses associated with conventional surgery. *Journal of Endovascular Therapy*.

[28] Hinchliffe RJ, Bruijstens L, MacSweeney STR, Braithwaite BD (2006). A randomised trial of endovascular and open surgery for ruptured abdominal aortic aneurysm-results of a pilot study and lessons learned for future studies. *European Journal of Vascular and Endovascular Surgery*.

[29] Tilney NL, Bailey GL, Morgan AP (1973). Sequential system failure after rupture of abdominal aortic aneurysms: an unsolved problem in postoperative care. *Annals of Surgery*.

[30] Sprung J, Levy PJ, Tabares AH, Gottlieb A, Schoenwald PK, Olin JW (1998). Ischemic liver dysfunction after elective repair of infrarenal aortic aneurysm: incidence and outcome. *Journal of Cardiothoracic and Vascular Anesthesia*.

[31] Livingston EH, Passaro EP (1990). Postoperative ileus. *Digestive Diseases and Sciences*.

[32] Avrahami RJ, Cohen JD, Haddad M, Singer P, Zelikovski A (1999). Gastric emptying after elective abdominal aortic aneurysm surgery: the case for early postoperative enteral feeding. *European Journal of Vascular and Endovascular Surgery*.

[33] Arya N, Sharif MA, Lau LL (2010). Retroperitoneal approach to abdominal aortic aneurysm repair preserves splanchnic perfusion as measured by gastric tonometry. *Annals of Vascular Surgery*.

[34] Mentec H, Dupont H, Bocchetti M, Cani P, Ponche F, Bleichner G (2001). Upper digestive intolerance during enteral nutrition in critically ill patients: frequency, risk factors, and complications. *Critical Care Medicine*.

[35] Nguyen AT, de Virgilio C (2007). Transperitoneal approach should be considered for suspected ruptured abdominal aortic aneurysms. *Annals of Vascular Surgery*.

[36] Kreis ME (2006). Postoperative nausea and vomiting. *Autonomic Neuroscience*.

[37] Junnarkar S, Lau LL, Edrees WK (2003). Cytokine activation and intestinal mucosal and renal dysfunction are reduced in endovascular AAA repair compared to surgery. *Journal of Endovascular Therapy*.

[38] Vedder NB, Winn RK, Rice CL, Chi EY, Arfors K-E, Harlan JM (1988). A monoclonal antibody to the adherence-promoting leukocyte glycoprotein, CD18, reduces organ injury and improves survival from hemorrhagic shock and resuscitation in rabbits. *Journal of Clinical Investigation*.

[39] Yassin MM, Barros D’Sa AAB, Parks TG (1998). Lower limb ischaemia-reperfusion injury causes endotoxaemia and endogenous antiendotoxin antibody consumption but not bacterial translocation. *British Journal of Surgery*.

[40] Wehrens XH, Rouwet EV, oude Egbrink MGA, Slaaf DW, Ramsay G (2002). Effects of experimental lower-limb ischaemia-reperfusion injury on the mesenteric microcirculation. *British Journal of Surgery*.

[41] Veith FJ, Tanquilut EM, Ohki T (2003). Conservative observational management with selective delayed repair for large abdominal aortic aneurysms in high risk patients. *Journal of Cardiovascular Surgery*.

[42] Greenberg RK, Chuter TAM, Lawrence-Brown M, Haulon S, Nolte L (2004). Analysis of renal function after aneurysm repair with a device using suprarenal fixation (zenith AAA endovascular graft) in contrast to open surgical repair. *Journal of Vascular Surgery*.

[43] Alsac JM, Desgranges P, Kobeiter H, Becquemin J-P (2005). Emergency endovascular repair for ruptured abdominal aortic aneurysms: feasibility and comparison of early results with conventional open repair. *European Journal of Vascular and Endovascular Surgery*.

[44] Hechelhammer L, Lachat ML, Wildermuth S, Bettex D, Mayer D, Pfammatter T (2005). Midterm outcome of endovascular repair of ruptured abdominal aortic aneurysms. *Journal of Vascular Surgery*.

[45] Peppelenbosch N, Geelkerken RH, Soong C (2006). Endograft treatment of ruptured abdominal aortic aneurysms using the Talent aortouniiliac system: an international multicenter study. *Journal of Vascular Surgery*.

[46] Sadat U, Boyle JR, Walsh SR, Tang T, Varty K, Hayes PD (2008). Endovascular vs open repair of acute abdominal aortic aneurysms-A systematic review and meta-analysis. *Journal of Vascular Surgery*.

[47] Greco G, Egorova N, Anderson PL (2006). Outcomes of endovascular treatment of ruptured abdominal aortic aneurysms. *Journal of Vascular Surgery*.

[48] Gerassimidis TS, Papazoglou KO, Kamparoudis AG (2005). Endovascular management of ruptured abdominal aortic aneurysms: 6-Year experience from a Greek center. *Journal of Vascular Surgery*.

[49] Lee WA, Hirneise CM, Tayyarah M, Huber TS, Seeger JM (2004). Impact of endovascular repair on early outcomes of ruptured abdominal aortic aneurysms. *Journal of Vascular Surgery*.

